# Ethnic differences in the lifestyle behaviors and premature coronary artery disease: a multi-center study

**DOI:** 10.1186/s12872-023-03192-0

**Published:** 2023-03-29

**Authors:** Media Babahajiani, Ehsan Zarepur, Alireza Khosravi, Noushin Mohammadifard, Feridoun Noohi, Hasan Alikhasi, Shima Nasirian, Seyed Ali Moezi Bady, Parisa Janjani, Kamal Solati, Masoud Lotfizadeh, Samad Ghaffari, Elmira Javanmardi, Arsalan Salari, Mahboobeh Gholipour, Mostafa Dehghani, Mostafa Cheraghi, Ahmadreza Assareh, Habib Haybar, Seyedeh Mahdieh Namayandeh, Reza Madadi, Javad Kojuri, Marjan Mansourian, Nizal Sarrafzadegan

**Affiliations:** 1 Student Research Committee, Vice Chancellor for Research and Technology, Kurdistan University of Medical Sciences, Sanandaj, Iran; 2 Interventional Cardiology Research Center, Cardiovascular Research Institute, Isfahan University of Medical Sciences, Isfahan, Iran; 3 Department of Cardiology, Medicine School, Isfahan University of Medical Sciences, Isfahan, Iran; 4 Hypertension Research Center, Cardiovascular Research Institute, Isfahan University of Medical Sciences, Isfahan, Iran; 5 The Iranian Network of Cardiovascular Research (INCVR), Isfahan, Iran; 6 Isfahan Cardiovascular Research Center, Cardiovascular Research Institute, Isfahan University of Medical Sciences, Isfahan, Iran; 7 Shahid Rajaie Cardiovascular, Medical and Research Center, Iran University of Medical Sciences, Tehran, Iran; 8 Heart Failure Research Center, Cardiovascular Research Institute, Isfahan University of Medical Sciences, Isfahan, Iran; 9 Pediatric Cardiovascular Research Center, Cardiovascular Research Institute, Isfahan University of Medical Sciences, Isfahan, Iran; 10 Cardiovascular Diseases Research Center, Birjand University of Medical Sciences, Birjand, Iran; 11 Clinical Research Development Unit, Imam Reza Hospital, Birjand University of Medical Sciences, Birjand, Iran; 12 Cardiovascular Research Center, Health Institute, Kermanshah University of Medical Sciences, Kermanshah, Iran; 13 Department of Psychiatry, Shahrekord University of Medical Sciences, Shahrekord, Iran; 14 Social Determinants of Health Research Center, Shahrekord University of Medical Sciences, Shahrekord, Iran; 15 Cardiovascular Research Center, Tabriz University of Medical Sciences, Tabriz, Iran; 16 Department of Cardiovascular Medicine, Amiralmomenin Hospital, Maragheh University Medical Sciences, Maragheh, Iran; 17 Department of cardiology, Healthy heart research center, Heshmat hospital, School of medicine, Guilan University of Medical Sciences, Rasht, Iran; 18 Department of Cardiology, Healthy Heart Research Center, Heshmat Hospital, School of Medicine, Guilan University of Medical Sciences, Rasht, Iran; 19 Department of Cardiovascular research Center, Shahid Rahimi Hospital, Lorestan university of Medical Science, Khorramabad, Iran; 20 Department of Cardiovascular Research Center, Shahid Rahimi Hospital, Lorestan university of Medical Science, Khorramabad, Iran; 21 Atherosclerosis Research Center, Ahvaz Jundishapur University of Medical Sciences, Ahvaz, Iran; 22 Yazd Cardiovascular Research Center, Shahid Sadooghi University of Medical Science, Yazd, Iran; 23 Zanjan University of Medical Sciences, Zanjan, Iran; 24 Clinical Education Research Center, Shiraz University of Medical Sciences, Shiraz, Iran; 25 Isfahan Cardiovascular Research Center, Cardiovascular Research Institute, Isfahan University of Medical Sciences, Isfahan, Iran; 26 Faculty of Medicine, School of Population and Public Health, University of British Columbia, Vancouver, Canada

**Keywords:** Premature coronary artery disease, Ethnic group, Lifestyle, Iran

## Abstract

**Background:**

Diverse ethnic groups that exist in Iran may differ regarding the risk factors such as hypertension, hyperlipidemia, dyslipidemia, diabetes mellitus, and family history of non-communicable disease. Premature Coronary Artery Disease (PCAD) is more endemic in Iran than before. This study sought to assess the association between ethnicity and lifestyle behaviors in eight major Iranian ethnic groups with PCAD.

**Methods:**

In this study, 2863 patients aged ≤ 70 for women and ≤ 60 for men who underwent coronary angiography were recruited in a multi-center framework. All the patients’ demographic, laboratory, clinical, and risk factor data were retrieved. Eight large ethnicities in Iran, including the Farses, the Kurds, the Turks, the Gilaks, the Arabs, the Lors, the Qashqai, and the Bakhtiari were evaluated for PCAD. Different lifestyle components and having PCAD were compared among the ethnical groups using multivariable modeling.

**Results:**

The mean age of the 2863 patients participated was 55.66 ± 7.70 years. The Fars ethnicity with 1654 people, was the most subject in this study. Family history of more than three chronic diseases (1279 (44.7%) was the most common risk factor. The Turk ethnic group had the highest prevalence of ≥ 3 simultaneous lifestyle-related risk factors (24.3%), and the Bakhtiari ethnic group had the highest prevalence of no lifestyle-related risk factors (20.9%). Adjusted models showed that having all three abnormal lifestyle components increased the risk of PCAD (OR = 2.28, 95% CI: 1.04–1.06). The Arabs had the most chance of getting PCAD among other ethnicities (OR = 2.26, 95%CI: 1.40–3.65). While, the Kurds with a healthy lifestyle showed the lowest chance of getting PCAD (OR = 1.96, 95%CI: 1.05–3.67)).

**Conclusions:**

This study found there was heterogeneity in having PACD and a diverse distribution in its well-known traditional lifestyle-related risk factors among major Iranian ethnic groups.

## Background

Coronary Artery Disease (CAD) is the most common heart-related non-communicable disease in industrialized countries. It is responsible for increasing deaths due to Cardiovascular Disease (CVD) worldwide [1, 2], affecting older people of all ethnicities and races [3–5]. Furthermore, the morbidity and mortality from CAD in patients with Premature Coronary Artery Disease (PCAD) (males < 55 years and females < 65 years) may have a devastating impact on the families of these patients [6, 7]. It is estimated that about 4–10% of individuals with documented CAD are premature [2, 8]. CAD-related deaths in Iran account for approximately 39.3% of the total deaths per year [9]. According to the GBD report in 2015, Iran was one of the countries with the most CVD rate in the world (9000 cases of CVD per 100 000 persons) [10, 11]; moreover, the prevalence of CAD and its risk factors is higher than in western countries [12]. Ardabil, North West province of Iran, with 50.1% has the most prevalent CVD in Iran [13]; on the contrary, the prevalence of CVD in the south of Iran reported was 10.4% [14]. In 2020, a study predicted mean 10-year CVD development as 16.4% [14], which makes it vital to conduct more studies. The majority of the burden entailed by CAD is related to modifiable risk factors. Various studies have proved that modifiable lifestyle factors such as smoking cessation, exercise, and a healthy diet can help reduce the risk of CAD [15–18]. Moreover, previous studies have shown that adherence to a healthy lifestyle, including a combination of the factors above, has reduced the incidence of CAD by approximately 40 − 45% [19, 20].

Different ethnicities may experience different CAD severities due to lifestyle factors [3–5, 21–24]. Iran, a multiethnic Middle Eastern country with diverse cultures, traditions, habits, and diets [25], is susceptible to CAD risk factors. The Farses, the Kurds, the Turks, the Gilaks, the Arabs, the Lors, the Qashqaei, and the Bakhtiari are Iran’s main ethnic groupings.

However, no information has been published on the potential relationship of life-related risk factor diversity with the risk of premature CAD in different ethnicities. Thus, the present study aimed to analyze, for the first time, whether the lifestyle is associated with premature CAD in the Iranian ethnic groups.

## Methods

### Design and subjects

This was a case-control study named *Iran-premature coronary artery disease* (I-PAD) study started in 2020, and is still ongoing on Iranian patients. Patients underwent coronary angiography with different ethnicities in Iran; including the Farses, the Kurds, the Turks, the Gilaks, the Arabs, the Lors, the Qashqaei, and the Bakhtiari. The patients were registered from hospitals with catheterization laboratories in different cities. The angiography databank at a multi-center framework was utilized for the current study. The demographic, laboratory, clinical, and risk factor data of all patients, who underwent coronary angiography were collected by trained physicians. Patients completed questionnaires that included information such as demographic, type of ethnicity, metabolic variables, lifestyle behaviors, and family history of illness [26].

### Inclusion and exclusion criteria

Inclusion criteria included patients who underwent coronary angiography, age ≤ 65 and ≤ 55 years for male and female, respectively. Patients were labeled as having CAD if they had at least 75% or more of a single coronary artery obstruction or 50% or more of the left main coronary artery. Patients labeled as not having PCAD if had normal arteries. Patients with a registered history of coronary artery diseases such as balloon angioplasty, Coronary Artery Bypass Graft (CABG), or Percutaneous Coronary artery Intervention (PCI) were excluded from the study. Hence, 3033 patients were eligible to enter the study, out of which 170 patients were excluded from the study due to incorrect information.

### Measures

Lifestyle-related factors, which are a combination of three variables; smoking, physical activity, and diet, were collected through the questionnaires.

Eating habits were measured using the validated semi-qualitative Food Frequency Questionnaire (FFQ). For dietary scoring purposes, 12 food categories were predetermined, and the consumption frequency for each food category in each patient was measured. Healthy food groups included fruits, vegetables, dairy products, non-hydrogenated vegetable oils, legumes, nuts, and white meat; unhealthy food groups included hydrogenated vegetable oils, red meat, processed meat, all grains, Pizza, and sweets. Patients with the highest healthy food consumption were scored one, and those with the highest unhealthy food consumption scored zero for that category. Diet in patients was scored from zero to 12, those with a score of eight or higher being classified as having a healthy diet and receiving a score of one. On the other hand, those with a score of less than eight were classified as having an unhealthy diet receiving a score of zero [27].

For the smoking variable, those who quit smoking six months ago or never smoked at all were defined as low-risk and received a score of one. Moreover, for the physical activity variable, those who had exercised for an average of at least 30 min of daily exercise with moderate or high intensity were classified as low-risk and received a minimum score [27].

The lifestyle variable combined three variables (smoking, physical activity, and diet), and it was scored as a value between zero to three for each patient (zero for having no abnormal status of these three variables, one for patients who had one abnormal lifestyle component and two for patients with two abnormal components). Patients with all abnormal components scored as three. Individuals with a score of one or higher were classified as having an unhealthy lifestyle.

The data on family history gathered were the history of first-degree relatives with definite chronic disease (family history of CVD, hyperlipidemia, diabetes mellitus, and hypertension), hypertension (defined by current use of antihypertensive medication or history of blood pressure > 140⁄95 mm Hg), and diabetes mellitus (Self-reported). Body mass index (BMI) was calculated as weight in kilograms divided by the square of height in meters (kg ⁄m^2^). Obesity was defined as BMI ≥ 30 kg⁄m^2^, hyperlipidemia (defined by total cholesterol ≥ 240, LDL cholesterol ≥ 130, Non-HDL cholesterol triglycerides ≥ 150, and HDL cholesterol < 40) [28]. Further details on the study method are described in depth in the I-PAD methodology Sect. [26].

## Statistical analysis

Patients were categorized into eight groups according to different ethnicities. The continuous variables were described with means and standard deviations (SDs), and the categorical variables were expressed as frequencies with percentages among the ethnic groups. Chi-square tests were used to compare the distribution of different categorical characteristics and ethnicities. One-way Analysis of Variance (ANOVA) or Kruskal-Wallis test was used to compare the level of continuous factors in different ethnicities. All clinically important variables were considered using stepwise logistic regression analyses. Adjusted logistic regression was performed to evaluate the relationship between PCAD as the dependent variable, and the risk factors such as sex, age, BMI, hyperlipidemia, dyslipidemia, diabetes mellitus, and family history of chronic disease, and dummy variables for the ethnic groups as the independent variables. The Fars was determined as the reference group among all ethnicities, due to its dominant lifestyle in Iran. Next, we applied four separate multivariable models for each ethnic group. Akaike information criterion (AIC) was used to compare different models. P-Value ≤ 0.05 was considered statistically significant. All the data analyses were conducted by SPSS 22.0 (IBM Corp, Armonk, NY, USA).

## Results

In this study, among 2863 patients (1556 male (54.3%)) with a mean age of 55.66 ± 7.70 who underwent coronary angiography, 1756 (61.3%) had a positive result for PCAD. The distribution of study subjects from different ethnicities were the Farses (1654 (57.8%)), the Turks (103 (3.6%)), the Gilaks (238 (8.3%)), the Kurds (364 (12.7%)), the Arabs (90 (3.1%)), the Lors (71 (2.5%)), the Qashqaei (127 (4.4%)), and the Bakhtiari (196 (6.8%)).

Table 1 shows the patient characteristics of the study sample. Among all patients, according to their angiography results, the most common risk factor was family history of more than three chronic diseases (1279 (44.7%), followed by hyperlipidemia (1148 (40.1%)), hypertension (1013 (35.4%)), and diabetes mellitus (722 (25.2%)). Out of these risk factors, the prevalence of hyperlipidemia and family history of chronic disease was significantly different among different ethnic groups (p-value < 0.05). The highest prevalence of diabetes mellitus and hyperlipidemia were presented in the Gilak ethnicity, with values of 80 (33.6%) and 116 (48.7%), respectively. In addition, the Lor had the highest prevalence of family history of chronic diseases (51.2%) (Table 1).

**Table 1:** **Characteristics of Study Patients between Different Ethnicity Groups**

	Total N = 2863	Fars N = 1654	Turk N = 103	Gilak N = 238	Kurd N = 364	Arab N = 90	Lor N = 71	Ghashghaei N = 127	Bakhtiari N = 196	P-value
**Positive Angiography results (abnormal) n (%)**	1756(61.3%)	1008(60.9%)	72(69.9%)	172(72.3%)	192(52.7%)	62(68.9%)	48(67.6%)	71(55.9%)	124(63.3%)	< 0.001
**Age (yr.)**	55.66 ± 7.70	55.83 ± 7.37	54.35 ± 8.98	57.21 ± 7.83	55.29 ± 7.99	53.67 ± 8.05	54.25 ± 10.76	56.00 ± 7.06	55.08 ± 7.70	0.003
**Sex (Male) n (%)**	1556(54.3%)	951 (57.5%)	71(68.9%)	109(45.8%)	157(43.1%)	36 (40.0%)	41 (57.7%)	66 (52.0%)	66 (52.0%)	< 0.001
**BMI ≥ 30n (%)**	891 (31.1%)	503 (30.4%)	33 (32.0%)	65 (27.3%)	131(36.0%)	32 (35.6%)	28 (39.4%)	40 (31.5%)	55 (28.1%)	0.191
**Diabetes n (%)**	722 (25.2%)	422 (25.5%)	33 (32.0%)	80 (33.6%)	72 (19.8%)	29 (32.2%)	14 (19.7%)	18 (14.2%)	47 (24.0%)	< 0.001
**Hypertension n (%)**	1013(35.4%)	567 (34.3%)	42 (41.2%)	100 (42.2%)	137(37.6%)	40 (44.4%)	23 (32.4%)	40 (31.5%)	58 (29.6%)	0.051
**Hyperlipidemia n (%)**	1148(40.1%)	677 (40.9%)	50 (48.5%)	116 (48.7%)	135(37.1%)	34 (37.8%)	20 (28.2%)	39 (30.7%)	68 (34.7%)	0.002
**Family History of chronic disease ≥ 3 n (%)**	1279(44.7%)	772(46.7%)	51(49.5%)	65(27.3%)	175(48.1%)	35(38.9%)	37(52.1%)	61(48.0%)	76(38.8%)	< 0.001

*BMI = body mass index*

**Fig. 1:**
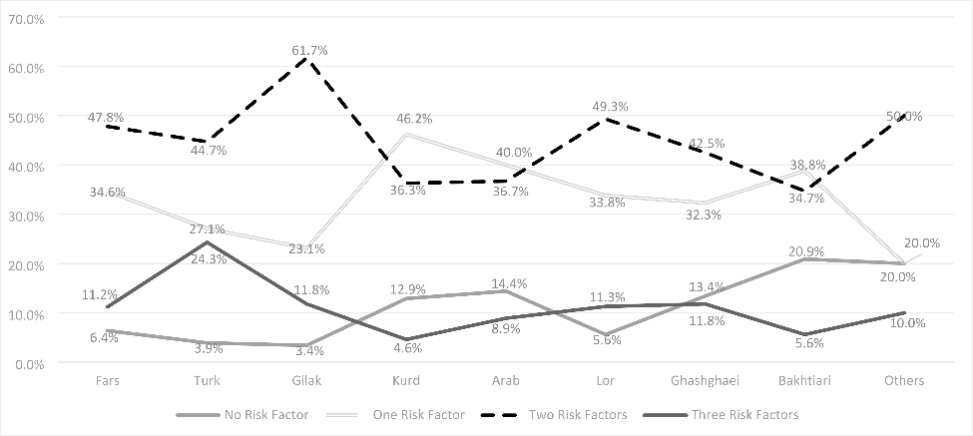
*Prevalence of Different Numbers of Unhealthy Lifestyle Components across Different Ethnic Groups*

Figure 1 shows that among different ethnicities, the Kurd, the Gilak, and the Turk ethnic groups had the highest prevalence of one, two, and three lifestyle risk factors (unhealthy dietary intake, smoking habit, and low physical activity), respectively.

Multiple logistic regression was conducted to test the relationship between PCAD and ethnicity, and the relationship between PCAD and lifestyle. The odds ratios were calculated in the adjusted and unadjusted models (Table 2).

**Table 2:** The Association of, Lifestyle Component and Ethnicity with PCAD according to both Adjusted and Un-Adjusted model

Adjusted Model^*^					Un-Adjusted Model				
Lifestyle	p-Value	Odds Ratio	(95% CI)	Lifestyle		p-Value		Odds Ratio	(95% CI)
No risk factor	Reference				No risk factor		Reference		
Having one abnormal component	0.102	1.27	(0.95- 1.71)	Having one abnormal component		0.117		1.25	(0.95- 1.66)
Having two abnormal component	0.351	1.14	(0.86- 1.52)	Having two abnormal component		0.388		1.29	(0.85- 1.48)
All three component were abnormal	< 0.001	2.28	(1.04–1.06)	All three component were abnormal		< 0.001		2.023	(1.41–2.86)
**Adjusted Model** ^*^					**Un-Adjusted Model**				
**Ethnicity**	**p-Value**	**Odds Ratio**	**(95% CI)**	**Ethnicity**		**p-Value**		**Odds Ratio**	**(95% CI)**
Fars	Reference			Fars		Reference			
Turk	0.01	1.81	(1.15–2.83)	Turk		0.071		1.48	(0.966- 2.294)
Gilak	0.005	1.57	(0.94- 1.58)	Gilak		0.001		1.67	(1.23–2.25)
Kurd	0.013	0.74	(0.94- 1.15)	Kurd		0.004		0.715	(0.56- 0.90)
Arab	0.001	2.26	(1.40–3.65)	Arab		0.133		1.41	(0.89- 0.2.241)
Lor	0.072	1.62	(0.95- 2.76)	Lor		0.261		1.33	(0.806 − 2.220)
Ghashghaei	0.13	0.74	(0.51- 1.09)	Ghashghaei		0.264		0.813	(0.565 − 1.16)
Bakhtiari	0.82	1.04	(1.75–1.43)	Bakhtiari		0.528		1.104	(0.812 − 1.5)

^*^Adjusted for sex, age, BMI, hyperlipidemia, dyslipidemia, diabetes mellitus, and family history of chronic disease

Having all three abnormal lifestyle components significantly increased the risk of PACD by 128 and 102% in both adjusted and unadjusted logistic regression, respectively. Furthermore, the results of the adjusted model for the relationship between PCAD and ethnicities showed that the highest chance of getting PCAD was seen in the Arab (OR = 2.26, 95% CI: 1.40–3.65) followed by the Turks (OR = 1.81, 95%CI: 1.15–2.83) and the Gilaks (OR = 1.57, 95% CI: 0.94- 1.58); in contrast, the chance of getting PCAD was significantly lower in the Kurds rather than the Farses (OR = 0.74, 95% CI: 0.94-0.1.15).

Table 3 demonstrates more details on the relationship between unhealthy lifestyle behaviors (having at least one abnormal component) and PCAD among different ethnicities. In the Lors and the Farses ethnicities, an unhealthy lifestyle increased the risk of PCAD significantly in all models. The unhealthy lifestyle in the Bakhtiari and the Kurds showed a significant relationship with getting PCAD in model one, two, and three (p-Value < 0.05).

**Table 3:** Association between PCAD and Unhealthy Lifestyle Behaviors (at least one abnormal component of smoking, low physical activity, and unhealthy diet) in Different Ethnicities

Ethnicities	Crude Model ^a^				Model One ^b^			Model Two ^c^			Model three ^d^		
	OR (95% CI)	p-Value	AIC	OR (95% CI)		p-Value	AIC	OR (95% CI)	p-Value	AIC	OR (95% CI)	p-Value	AIC
Fars	1.98(1.40–2.79)	< 0.001	2216.8	2.25(1.58–3.19)		< 0.001	2188.8	2.11(1.48–3.02)	< 0.001	2130.1	2.09(1.46–2.99)	< 0.001	2128.5
Turk	2.00(0.67-5.93)	0.211	126.43	1.10(0.312 − 3.89)		0.881	111.26	1.28(0.33-4.91)	0.723	119.18	1.24(0.320 − 4.81)	0.754	120.87
Gilak	1.23(0.55-2.77)	0.615	283.91	1.61(0.70-3.72)		0.265	274.26	1.51(0.62 − 3.700)	0.346	260.46	2.13(1.02–4.44)	0.044	258.06
Kurd	1.39(0.78-2.48)	0.257	505.76	1.81(0.100-3.297)		0.051	445	1.92(1.03–3.56)	0.039	437.34	1.96(1.05–3.67)	0.035	433.2
Arab	1.69(0.64-4.43)	0.287	115.23	1.629(0.57-4.61)		0.358	111.45	1.78(0.566 − 5.62)	0.323	118	1.78(0.563 − 5.61)	0.327	119.45
Lor	3.20(0.1.11–9.21)	0.031	90.18	3.11(1.06–9.11)		0.038	90.59	4.28(1.24–14.73)	0.021	93.48	4.16(1.19–14.46)	0.025	94.68
Ghashghaei	1.58(0.74-3.39)	0.241	126.43	2.04(0.86-4.87)		0.106	126.82	2.05(0.81-5.19)	0.127	131.38	1.91(0.741 − 4.90)	0.181	133.19
Bakhtiari	1.31(0.719 − 2.40)	0.375	258.59	2.15(1.02–4.56)		0.045	250.53	2.27(1.04–4.92)	0.039	254.19	2.23(1.03–4.86)	0.043	256.18

*CI = Confidence Interval, OR = Odds Ratio*

*“* ^*a*^ *” = Unhealthy Lifestyle*

*“* ^*b*^ *” = Model “a” ” and additionally adjusted Sex and Age*

*“* ^*c*^ *” = Model “b” and additionally adjusted for body mass index, hypertension, hyperlipidemia, dyslipidemia, diabetes mellitus*

*“* ^*d*^ *” = Model “c” and additionally adjusted for family history of cardiovascular disease, family history of hyperlipidemia, family history of diabetes mellitus, family history of hypertension*

*Risk factors = unhealthy dietary intake, smoking habits, and low physical activity*

Based on the full adjusted model results, the most significant increase in odds ratio was for the Lors ethnicity (OR = 4.156, 95% CI: 1.990–14.460), and the least significant increase was related to the Kurds (OR = 1.964, 95% CI: 1.050–3.671).

## Discussion

The current study examined the relationship between PCAD and lifestyle risk factors expressed as diet, physical activity, and smoking while considering ethnicity. The prevalence of PCAD among the Gilaks and the Turks ethnicities was significantly higher than in other ethnic groups and considerably lower in the Kurd ethnic group. Results showed that the prevalence of three and two simultaneous modifiable risk factors was significantly higher in the Turks and the Gilaks, respectively, compared with other ethnicities. At the same time, the Kurds had the lowest modifiable lifestyle risk factors.

We designed a logistic regression model to adjust the risk factors according to the observed differences between ethnicities. Patients with all three lifestyle risk factors had two times more chance of developing PCAD than others with no lifestyle risk factors. Furthermore, the full-adjusted model showed that the Lors and the Kurds ethnic groups had a higher and lower increase in the odds ratio of PCAD, respectively. As it is visible, the differences are still considerable.

Various studies have confirmed the relationship between ethnicity and cardiovascular diseases. They have also considered the importance of knowing the characteristics of different ethnicities to reduce racial and ethnic differences in heart disease [22, 29]. In a study in Kazakhstan, Zea-Vera et al. investigated this relationship. After adjusting for the traditional risk factors of cardiovascular diseases and the quality of life index, they found that the prevalence of this disease is higher in Russians than in Kazakhs [30].

In Spain, a cohort study from the University of Navarre called the *Seguimiento Universidad de Navarra (SUN)* cohort found that participants with better HLS scores (healthy lifestyle scores) had a significant and inverse relationship with the risk of primary cardiovascular events [31]. Moreover, Tran et al. showed that CVD rates were higher in Norwegians than in other ethnic minorities [32].

The present study, which examined most ethnicities in Iran with acceptable sample size, also provides results confirming the relationship between ethnicity and PCAD.

Our results also revealed that the chance of PCAD in the Arabs, followed by the Turks and the Gilaks, was significantly higher than in the Farses ethnicity. In contrast, the chance of PCAD was significantly lower in Kurds than in the Fars ethnicity, highlighting the characteristics of the different lifestyles associated with these ethnicities.

The Kurds are the third most populous ethnic group in Iran. The major Kurd ethnic groups in Iran live in the Zagros Mountains near the Turkey and Iraq borders. They are mostly residents of the western provinces of Iran, such as Kurdistan, Kermanshah, and Ilam. The mountainous habitat, along with dietary habits, has been able to influence the lifestyles of the people of this region and reduce the risk of PCAD compared with the Farses ethnicity, who are the common ethnicity of Iran [25, 33] and mostly live in the central regions of Iran. In contrast, the Arab ethnicity in Iran, who mostly live in southern provinces such as Khuzestan, Bandar Abbas, and cities along the coasts of the Persian Gulf, have a hot climate, with temperatures reaching 50 °C in dry seasons [34]. The sedentary lifestyle of the Arabs ethnicity, its hot climate, and unique cultural barriers to physical activity are essential indicators of the rapidly increasing prevalence of obesity in this population, as people prefer to spend more time at home and have less physical activity [35]. Some studies have shown that physical activity and diabetes mellitus are inversely related, and this relationship is much stronger in people with high genetic susceptibility. The fact that consanguineous marriages are common in Arab culture becomes as important as it concerns [36]. Therefore, due to the climatic, cultural, environmental, and regional factors, high-fat dietary habits and a sedentary lifestyle have led to a higher chance of developing PCAD than the Fars ethnicity. Nevertheless, the results of the study by Najafi et al. in the Persian cohort study of Guilan showed an inverse causality between (BMI) and physical activity. With increasing weight, the participants tended to have less physical activity [35]. Considering the lifestyle, high-calorie dietary habits, and high-carbohydrate foods could be the main reasons for the increase in obesity and overweight in the Gilak population in the north of the country. The evidence in the literature can highlight the high risk of PCAD in the Gilak men and women.

On the one hand, studies in this ethnic group have shown that the migration of the Gilak population from rural to urban areas due to economic problems has led to lifestyle changes and reduced physical activity. The average age of marriage for the Gilak women is about 20 years [37] and according to the results of Persian cohort studies in Guilan, 85.10% of women are married [38]. Since sex hormone-related factors may also play a role in weight gain [24], low marriage age can also contribute to the high PCAD risk in this ethnic group.

The study results regarding the Gilak and Turk ethnicity were in line with the results of Abbasi et al. They pointed out that the reasons for the high risk of PCAD in this ethnic group compared to the Fars group are a sedentary lifestyle, consumption of high-fat foods, and genetic factors [33].

Various studies performed in Azerbaijan have suggested that poor eating habits, diets high in carbohydrates and sodium, and inadequate consumption of healthy foods, and dairy products have increased the risk of cardiovascular disease in Turks. Moreover, the overall prevalence of smoking in these regions is higher than in Iran and the neighboring countries of Azerbaijan [39, 40]. Since lifestyle is a modifiable risk factor for PCAD, improving its components can reduce the risk of PCAD in the Fars, the Gilak, and the Bakhtiari populations.

## Strengths and Limitations

The present study is the first report to investigate the relationship between the lifestyle of different Iranian ethnic groups and PCAD. One of the study’s strengths is the large number of recruited patients representing most Iranian ethnicities. Another strength of the study is the recruitment of study patients, which excludes people of mixed ethnicities (ethnicities resulting from intermarriage). However, there were some measurement errors in this study, particularly in evaluating diet and physical activity due to self-reporting, which may have weakened the observed correlations. Second, there are other ethnicities in Iran, but their community is not large enough to be considered.

## Conclusion

This study found there was heterogeneity in having PACD and diverse distribution of its well-known traditional lifestyle-related risk factors among major Iranian ethnic groups.

The findings of this study add to our understanding of the role of lifestyle in different ethnic groups and may help health policymakers implement prevention programs in vulnerable ethnic groups.

## Data Availability

The datasets used and/or analyzed during the current study are available from the corresponding author on reasonable request.

## List of abbreviations

CAD: Coronary Artery Disease
CVD: Cardiovascular Disease
PCAD: Premature CAD
I-PAD: Iran-premature coronary artery disease)
CABG: Coronary Artery Bypass Graft
PCI: Percutaneous Coronary artery Intervention
FFQ: Food Frequency Questionnaire
SDs: Standard Deviations
ANOVA: Analysis of Variance
OR: Odds Ratio
CI: Confidence Interval
SUN: Seguimiento Universidad de Navarra
HLS: Healthy Lifestyle Scores
